# Fifty Years of Hydrosilylation in Polymer Science: A Review of Current Trends of Low-Cost Transition-Metal and Metal-Free Catalysts, Non-Thermally Triggered Hydrosilylation Reactions, and Industrial Applications

**DOI:** 10.3390/polym9100534

**Published:** 2017-10-20

**Authors:** Robin J. Hofmann, Matea Vlatković, Frank Wiesbrock

**Affiliations:** 1 Polymer Competence Center Leoben GmbH (PCCL), Roseggerstrasse 12, 8700 Leoben, Austria; robin.hofmann@pccl.at (R.J.H.); matea.vlatkovic@pccl.at (M.V.); 2 Institute for Chemistry and Technology of Materials, Graz University of Technology, NAWI Graz, Stremayrgasse 9, 8010 Graz, Austria

**Keywords:** hydrosilylation, catalysis, anti-Markovnikov addition, stereoselectivity

## Abstract

Hydrosilylation reactions, the (commonly) anti-Markovnikov additions of silanes to unsaturated bonds present in compounds such as alkenes and alkynes, offer numerous unique and advantageous properties for the preparation of polymeric materials, such as high yields and stereoselectivity. These reactions require to be catalyzed, for which platinum compounds were used in the initial stages. Celebrating the 50th anniversary of hydrosilylations in polymer science and, concomitantly, five decades of continuously growing research, hydrosilylation reactions have advanced to a level that renders them predestined for transfer into commercial products on the large scale. Facing this potential transfer, this review addresses and discusses selected current trends of the scientific research in the area, namely low-cost transition metal catalysts (focusing on iron, cobalt, and nickel complexes), metal-free catalysts, non-thermally triggered hydrosilylation reactions (highlighting stimuli such as (UV-)light), and (potential) industrial applications (highlighting the catalysts used and products manufactured). This review focuses on the hydrosilylation reactions involving alkene reactants.

## 1. Introduction

**Figure 1:**
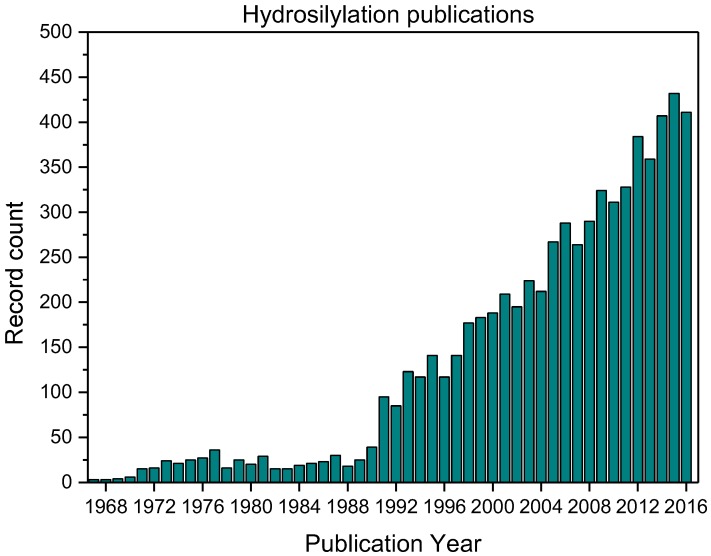
Publications on hydrosilylations in peer-reviewed journals, sorted per year.

**Figure 2:**
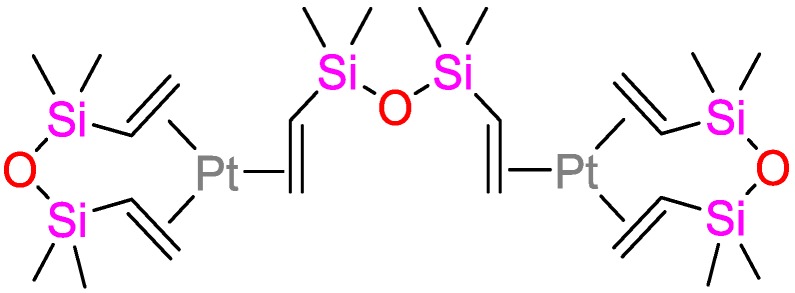
Karstedt’s catalyst.

**Figure 3:**
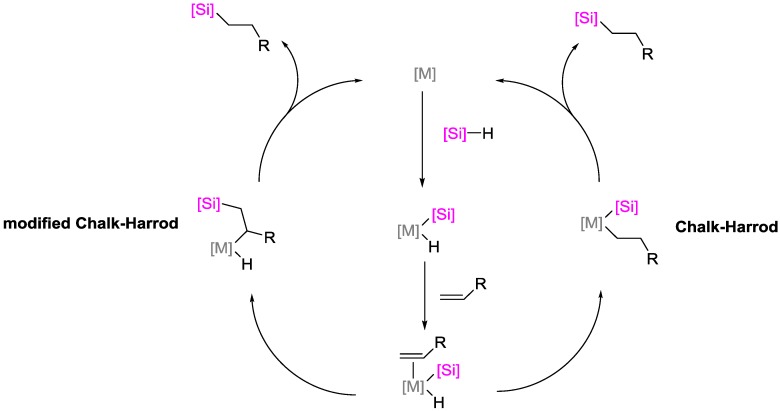
Chalk–Harrod mechanism and modified Chalk–Harrod mechanism for the hydrosilylation of alkenes.

Hydrosilylation reactions are widely implemented for the production of functional silanes and siloxanes. Preceded by the first report of hydrosilylations in 1947 (70 years ago) [1] and the first report of Speier’s platinum catalyst in 1957 (60 years ago) [2], the first application of a hydrosilylation reaction in polymer science dates back to 1967 (according to a Web of Science research; Figure 1) with an on-going research impetus at its 50th anniversary [3]. Concomitantly, academia and industry seek for price-efficient and durable catalysts that meet the increasing demand for silicon-based polymers. Targeted properties of such catalysts are their selectivity; activity, which is quantified by the turnover-frequency (TOF); and stability, which is defined by the turnover-number (TON). The majority of industrial hydrosilylation reactions are performed using the so-called Speier’s catalyst (H_2_PtCl_6_) and Karstedt’s catalysts (Figure 2) due to their high activity and selectivity. Hydrosilylation reactions catalyzed by platinum catalysts commonly follow Chalk–Harrod and modified Chalk–Harrod mechanisms (Figure 3), rendering side-reactions possible that yield unfavorable by-products and, correspondingly, lower the yield of the targeted product(s) and increase the production costs. Due to its low abundance, platinum and the corresponding compounds are high-priced; hence, research for alternative platinum-free catalysts containing low-cost-transition metals and/or metal-organic compounds [4,5] is performed extensively.

Due to the increasing demand for silanes and siloxanes by industrial manufacturers, novel hydrosilylation methods [6,7] have been in the focus of research over the last years, particularly since the beginning of the millennium. In a comprehensive review article [6], Troegel and Stohrer have summarized the state-of-the-art of olefin hydrosilylation from an industrial point of view, focusing on the years 2000–2010. In recent years, further developments have been made in low-cost transition metal catalysis and non-metal catalysis, triggered hydrosilylation, and novel applications of silanes and siloxanes, which will be presented in this review article. Hence, a section dedicated to novel platinum-based catalysts is excluded from this overview; the interested reader is referred to a current review article [8]. Instead, the main focus of this article is directed towards the hydrosilylation of olefin reactants; only selected examples of the hydrosilylation of other substrates will be discussed. Included in the section of hydrosilylation catalysis, triggered catalysis, such as photo-initiated hydrosilylation, will also be discussed. With special respect to the huge application potential, one section of this review is dedicated to the recent advances on hydrosilylated products for industrial applications. In summary, the main part of this review has been divided into four sections, namely on low-cost transition metal catalysts for hydrosilylation reactions (focusing on iron, cobalt, and nickel complexes; for a review on this research area exclusively, the reader is referred to a recent research article [9]), metal-free catalysts for hydrosilylation reactions, non-thermally triggered hydrosilylation reactions (highlighting stimuli such as (UV-)light, microwave irradiation, sonication, and electro-chemical activation), and industrial applications (highlighting the catalysts used and products manufactured). Notably, in the case of the transition metal catalysts, the terms “cheap” and “economic” always refer to the metal ion of the catalyst; the ligands, on the other hand, may require multi-step organic syntheses (with eventually low overall yields) and, concomitantly, may not necessarily be regarded as “cheap” and “economic”. 

## 2. Low-Cost Transition Metal Catalysts for Hydrosilylation

Platinum-based catalysis is abundantly used in the silicon industry and for hydrosilylation reactions themselves. Although prices for platinum have dropped by roughly 40% (from 58 to 32 k$ per kg) over the last five years [10], it remains a precious high-priced metal. Up to date, platinum catalysts are unmatched in catalytic activity, rendering them (still) the first choice for the hydrosilylation industry. To further reduce the costs of catalysis for hydrosilylation, it is necessary to replace platinum by, e.g., low-cost transition metals. This section of the review focuses on recent advances in hydrosilylation reactions catalyzed by low-cost metals such as iron, cobalt and nickel.

### 2.1. Iron Catalysts

With special respect to the pricing and potential for the large-scale production of consumer goods, Chirik et al. reported iron dinitrogen compounds with bis(imino)pyridine ligands [11] for the selective anti-Markovnikov additions of sterically hindered tertiary silanes to alkenes. These types of catalysts could be used under mild conditions: The crosslinking of commercially available silicone fluids could be accomplished at temperatures as low as 23 °C.

Nagashima et al. reported the application of iron complexes with *cyclo*-octatetra-1,3,5,7-enyl COT ligands as well as the ferrocene-based η^5^-3-methylpentadienyl and η^5^-2,4-dimethylpentadienyl ligands as precursors for the hydrosilylation of styrene [12]. As auxiliary ligands, bis(imino)pyridine ligands as well as alkylisocyanides such as adamantly isocyanide could be used, yielding iron-isocyanide species in the latter case, which were found to be efficient catalysts for the hydrosilylation reactions of styrene derivatives with, e.g., PhMe_2_SiH. In the temperature range from room temperature r.t. to 50 °C, the anti-Markovnikov products were selectively formed. The turn-over numbers TONs were higher than 100 and, for selected reactants investigated in this study, reached values as high as 5000.

**Figure 4:**
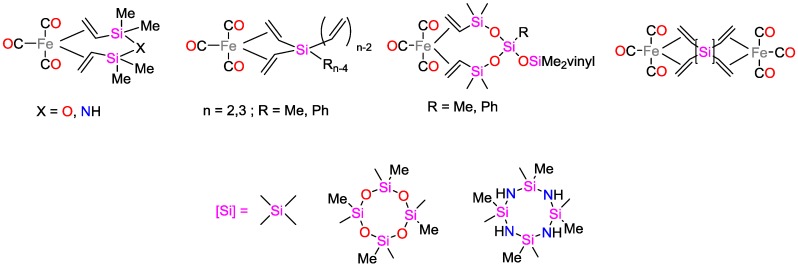
Iron complexes containing multivinyl-silicon ligands.

Mariciniec and coworkers [13] have employed a novel class of Fe complexes containing multivinyl-silicon ligands (Figure 4) for the hydrosilylation of poly(vinylsiloxane)s with poly(hydrosiloxane)s, yielding crosslinking polysiloxane networks. It was shown that these complexes efficiently catalyze the dehydrogenative silylation of tri(vinylsiloxane)s with tri(hydrosiloxane)s [14].

An iron-disilyl-dicarbonyl complex carrying a 1,2-bis(dimethylsilyl)benzyl ligand that (weakly) coordinated to the iron center in η^2^-(H–Si) was reported by Nagashima and coworkers [15]. This complex successfully catalyzed the hydrosilylation of alkenes (as well as the catalytic hydrogenation of alkenes and the hydrosilane-mediated reduction of carbonyl compounds). 

**Figure 5:**
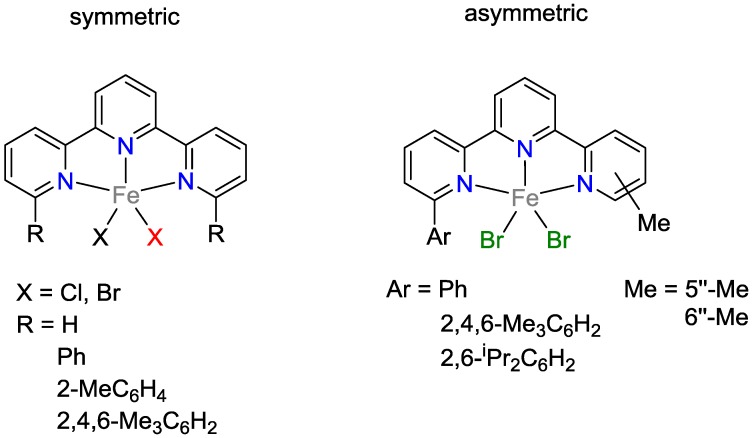
Terpyridine/iron complexes.

For the catalytic hydrosilylation of alkenes, Kamata and coworkers [16] used terpyridines and derivatives as ligands for the iron-catalyzed hydrosilylation (Figure 5). Upon the addition of NaH·BEt_3_, such iron compounds catalyzed the reaction of hex-1-ene with PhSiH_3_. It was also shown that non-substituted terpyridines did not exhibit any catalytic activity. Dibromo/iron complexes displayed higher activities (up to 95% yield) than dichloro/iron complexes. Interestingly, the change of the reactant from hex-1-ene to oct-1-ene on the one hand as well as the change from symmetric to asymmetric catalysts complexes on the other led to increased TONs of 1500. Notably, using higher amounts of the iron complex (up to 0.3 mol % Fe), the hydrosilylation reaction of oct-1-ene and PhSiH_3_ occurred as double addition, yielding the product PhSi(Oct)_2_H.

The application of Iron complexes with iminobipyridine ligands was also reported by Nakazawa et al. [17,18]. Preceded by activation with NaBHEt_3_, these catalysts were found to exhibit high catalytic activity for the hydrosilylation of terminal olefins with primary and secondary silanes [17]; several catalysts showed additional high activity for the hydrosilylation of terminal olefins with tertiary silanes as well [18].

**Figure 6:**
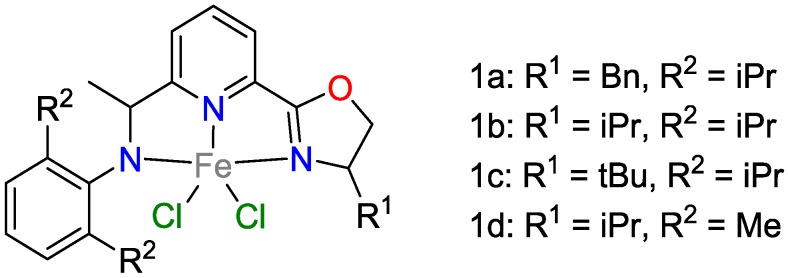
Hydrosilylation of 1,1-disubstituted aryl alkenes.

Chen and coworkers [19] achieved a key breakthrough in the hydrosilylation of alkenes using an iron-based catalyst system capable of regio- and enantioselective hydrosilylation of 1,1-disubstituted aryl alkenes. Among the dichloro/iron compounds tested (Figure 6), one catalyst (namely 1b) hydrosilylated 2-phenyl-but-1-ene with Ph_2_SiH_2_ in excellent yields of 92% and 94% enantiomeric excess *ee*. It was shown that bulkier ligands (namely 1c) decreased the catalytic activity (<5% yield), while lower steric hindrance (namely in 1d) reduced the enantioselectivity (62% *ee*). The system did not tolerate carbonyl groups such as ketones and aldehydes unless they were protected by acetal groups. The hydrosilylation of such protected carbonyl-functionalized alkenes yielded 78–92% with 79–93% *ee*.

Zuo and coworkers developed iminopyridine-oxazoline/iron complexes for the asymmetric hydrosilylation of ketones [20]. The complexes were applied in the reaction of 4-*^iso^* butylacetophenone with the secondary silane Ph_2_SiH_2_. It was shown that the catalyst with sterically demanding groups showed the highest yield of up to 97% with 93% *ee*.

**Figure 7:**
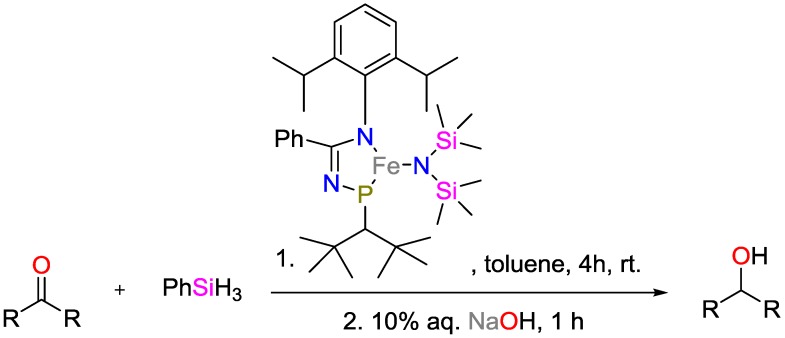
Hydrosilylation of ketones using an Fe^II^ *N*-phosphinoamidinate complex.

Ruddy and coworkers investigated the hydrosilylation of carbonyl compounds at room temperature r.t. [21], and developed an Fe^II^ *N*-phosphinoamidinate complex capable of catalyzing the hydrosilylation of ketones, aldehydes, and esters to yield secondary alcohols (Figure 7). The hydrosilylation of acetophenone with PhSiH_3_ proceeded with 99% yield. Notably, this iron compound catalyzed the reactions with 0.01–1 mol % Fe loading, while only the stochiometric 1 equivalent of the hydrosilane reductant needed to be added. Additionally, it was shown that the catalyst may be used along with 37 different substrates. To the best of our knowledge, this is the broadest range of substrates for one type of iron-based catalysts.

**Figure 8:**
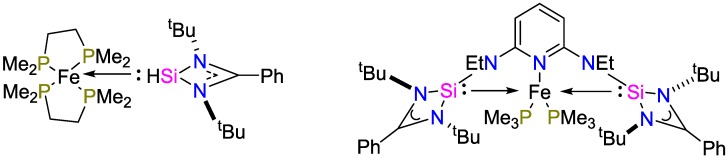
Fe complexes synthesized by Driess and coworkers.

By combined hydrosilylation experiments and density functional theory DFT calculations, Metaänen, Gallego and coworkers [22] investigated the hydrosilylation of a silylene/iron complex that was developed by Driess and coworkers [23]. According to these combined studies, a peripheral mechanism that does not directly involve the metal core was proposed for the hydrosilylation of ketones (Figure 8). The silyl group acts as Lewis acid coordinating the ketone, yielding a penta-coordinated silicon. After forming a Lewis pair, the hydrosilane coordinates to the carbonyl group. The hydrosilylated product is subsequently released after cleavage of the carbonyl-silicon-bond. 

### 2.2. Cobalt Catalysts

Due to a recent review by Sun and Deng on this topic [5], this section focuses on the most recent advances on hydrosilylation of alkenes and alkynes during the last years.

**Figure 9:**
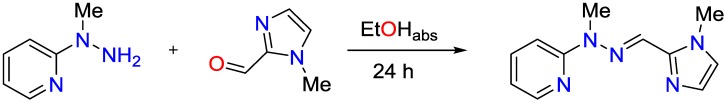
Synthesis of Schiff-base ligand.

Gorczynski and coworkers employed a new tridentade Schiff-base ligand for alkene hydrosilylation [24], which was formed by the condensation reaction of 2-(1-methylhydrazinyl) pyridine and 1-methyl-2-imidazolecarboxaldehyde (Figure 9). The catalyst itself was obtained by adding the ligand L to CoCl_2_·6H_2_O, yielding [CoLCl_2_]. From the screening of various hydrosilane substrates, it was revealed that, depending on the substrate, either hydrosilylation or dehydrogenative silylation may occur. It was shown that hydrosiloxanes mostly undergo dehydrogenative silylation, while phenyl-hydrosilanes are hydrosilylated in a reaction with terminal alkenes. However, triethylsilanes were not affected by the catalyst. 

Ibrahim and coworkers described the use of a bis(carbene) Co^I^/di-nitrogen complex for the hydrosilylation with tertiary silanes [25]. The mechanism using this catalyst followed the Chalk–Harrod path and exhibited high anti-Markovnikov selectivity for all substrates tested. The reaction of terminal alkenes with either Me_2_PhSiH or 1,1,1,3,5,5,5-heptamethyltrisiloxan in toluene showed high yields of up to 99% at r.t. In the reactions of alkenes with functional groups, the catalyst showed selective hydrosilylation of unsaturated alkene bonds, while functional groups such as unprotected alcohols, primary and tertiary amines as well as ketones and esters were tolerated.

Noda and coworkers demonstrated the hydrosilylation of alkenes with hydrosiloxanes and tertiary silanes [26]. They used pivaloyl Pv ligands for the generation of Co(OPv)_2_, allowing a reaction of styrene and α-methylstyrene with tertiary silanes in the presence of 1-adamantyl isocyanide. The resulting hydrosilylation reaction exhibited yields of up to 99% with TONs of up to 10,000. It was discovered that hydroalkoxysilanes enhanced the catalytic activity as co-catalysts, while they were less reactive towards the hydrosilylation reactions themselves. In one prominent example of this co-catalytic activity, the yield of the hydrosilylation of styrene with poly(dimethylsiloxane) (PDMS) was increased from 88% to 96% using (EtO)_2_MeSiH. The catalysts have been successfully used for the cross-linking of vinyl-functionalized PDMS and poly(dimethylhydroxysiloxane)s with yields up to 97%. 

**Figure 10:**
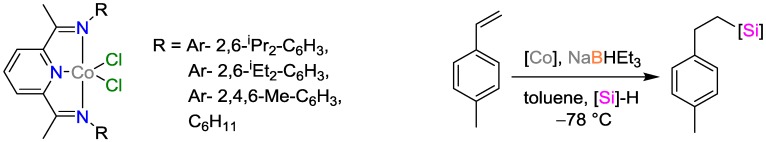
Cobalt catalyst for the hydrosilylation of alkenes.

Raya and coworkers reported the hydrosilylation of alkenes using a 2,6-bis(arylimino)-pyridine/CoCl_2_ complex (Figure 10) [27]. Employing NaH·BEt_3_ as activator, the complex showed high catalytic activity (up to 98% yield) towards the hydrosilylation of 4-methylstyrene with PhSiH_3_, Et_2_SiH_2_ and MePhSiH_2_ and moderate activity (50% yield) towards the hydrosilylation along with Ph_2_SiH_2_.

**Figure 11:**
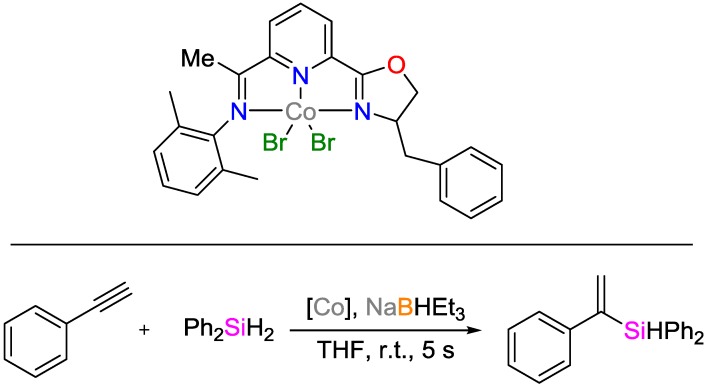
Cobalt catalyst system for alkyne hydrosilylation.

Guo and Lu developed a cobalt catalyst with an unmatched turn-over frequency TOF of 65,520 h^−1^ for the hydrosilylation of alkynes (Figure 11) [28]. Being highly chemo-, regio- and stereoselective, the catalyst showed tolerance towards a variety of functional groups such as alcohols and anilines, esters and ketones, as well as nitriles and amides, yielding conversion rates of up to 99% in the presence of NaH·BEt_3_. It was also shown that the hydrosilylation of alkynes can be followed by an anti-Markovnikov hydroboration of the vinylsilane to yield a hydroborated and hydrosilylated product. If this reaction is performed as one-pot reaction, a double-Markovnikov addition product is obtained. 

Lu et al. used complexes of CoCl_2_ with bulky iminiopyridine ligands for the sequential hydrosilylation and hydrogenation of alkynes, yielding chiral silane products [29]. Similar to the work described above, activation of the catalyst was accomplished by the addition of NaHBEt_3_. The syntheses, starting from reactants as simple as alkynes, silanes and hydrogen, were found to be highly regio- and enatioselective.

Mo and coworkers [30] reported the hydrosilylation of alkynes with a three-coordinated Co^I^ complex with bulky *N*-heterocyclic carbene ligands, aiming to improve the stereo- and regioselectivity of the hydrosilylation. The catalyst [Co(IAd)(PPh_3_)(CH_2_TMS)] (IAd = 1,3-di-adamantylimidazol-2-ylidene) was obtained from the alkylation of Co(IAd)(PPh_3_)Cl with LiCH_2_TMS. Reacting according to the modified Chalk-Harrod mechanism, the catalyst efficiently catalyzed the hydrosilylation yielding 98% *syn*-adducts of vinylsilanes. 

Ge and coworkers described the cobalt-catalyzed *Z*-selective anti-Markovnikov hydrosilylation of terminal alkynes (ethynylarenes as well as aliphatic alkynes) [31]. The catalysts were prepared from the reaction of equimolar amounts of cobalt acetate and pyridine-2,6-diimine ligands. The addition of phenol was required to suppress the isomerization of the *Z*-vinylsilanes and, consequently, maintain high *Z* selectivity. Huang et al. reported the *Z*-selective hydrosilylation of terminal alkenes with Ph_2_SiH_2_ by pincer cobalt complexes derived from CoCl_2_ [32]. Of special praise is the selective formation of *Z*-β-vinylsilanes in combination with the high tolerance of functional groups. Huang also reported the synthesis of α-vinylsilanes with high Markovnikov selectivity from the reaction of terminal alkynes with Ph_2_SiH_2_ using iminopyridine CoCl_2_ complexes [33]. Again, high selectivity could be observed concomitant with high functional group tolerance; the vinylsilanes could be reacted to germinal borosilanes in subsequent reactions.

**Figure 12:**
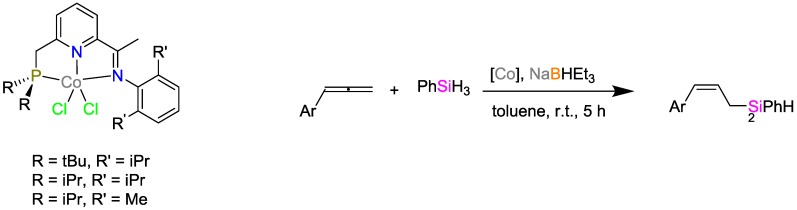
Cobalt catalyst for the hydrosilylation of allenes with high regio- and *Z*-selectivity.

The hydrosilylation of monosubstituted as well as 1,1-disubstituted allenes with high regio- and *Z*-selectivity by cobalt catalysts has been described by Huang, Ma, and coworkers (Figure 12) [34]. A pincer complex of the composition (^tBu^P^C^NN^iPr^)CoCl_2_ was found to deliver highest yields with respect to the regio- and *Z*-selectivity after activation by NaH·BEt_3_.

**Figure 13:**
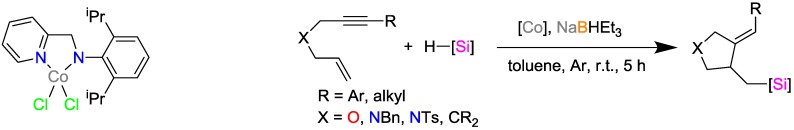
Cobalt catalyst for the combined hydrosilylation and cyclization of 1,6-enynes.

Lu et al. reported an iminopyridine complex of cobalt dichloride as catalyst for the combined hydrosilylation and cyclization of 1,6-enynes (Figure 13) [35]. This reaction could be scaled-up to the gram scale. A large variety of functional groups such as (among others) amines, esters, and free anilines were tolerated.

### 2.3. Nickel Catalysts

Srinivas and coworkers used commercially available Ni(acac)_2_ (acac: acetylacetonate) and derivatives to explore their catalytic activities in hydrosilylation [36]. It was demonstrated that, in the presence of NaH·BEt_3_ at r.t., the catalyst system showed catalytic activity towards the hydrosilylation of 1,3-dienes and α-alkenes giving yields from 75% to 85%. For the hydrosilylation of 1,3-dienes, the catalysts exhibited high 1,4-regioselectivity. In addition, the catalysts were tested for alkyne hydrosilylation, resulting in moderate yields of up to 64%. 

**Figure 14:**
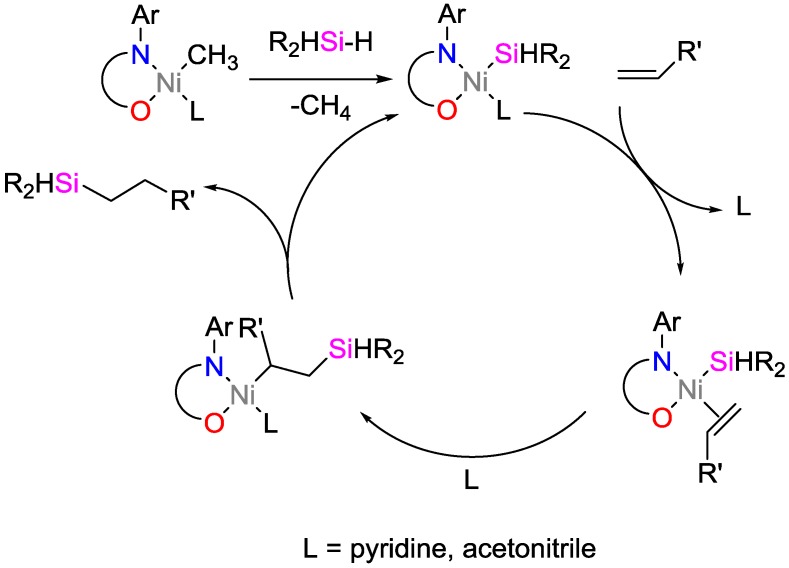
Suggested mechanism for the olefin hydrosilylation by (salicylaldiminato)Ni^II^ catalysts.

The group of Srinivas and coworkers investigated (salicylaldiminato)Ni^II^ catalysts and suggested a mechanism of th catalytic activity for the hydrosilylation of various olefins (Figure 14) [37]. The Ni complex exhibited high catalytic activity towards the hydrosilylation of secondary hydrosilanes with yields of up to 93% and high selectivity for mono-hydrosilylation.

**Figure 15:**
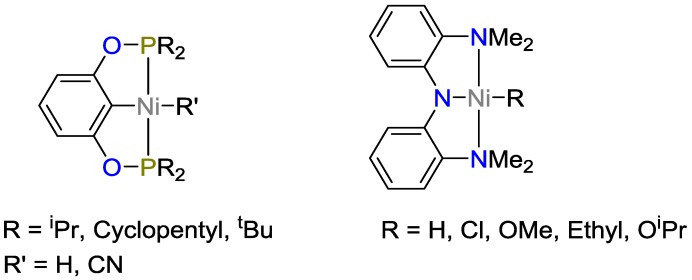
Ni-Pincer-Complex for hydrosilylation of: ketones and aldehydes (**Left**); and alkenes (**Right**).

Buslov and coworkers [38] reported the hydrosilylation of alkenes catalyzed by nickel-based pincer complexes (Figure 15). This type of complexes was found to be efficient (up to 93% yield) for the anti-Markovnikov hydrosilylation of terminal alkenes as well as for functionalized alkenes. The pincer complexes exhibited high catalytic activity towards secondary silanes with TOFs of 83,000 h^−1^ and TONs of 10,000, while the complex showed low activity towards primary and tertiary silanes. To explore the limits of this system, the catalysts were tested along various functional groups, showing that epoxides, aryl and alkyle bromides as well as primary and tertiary amides were tolerated. However, the catalysts showed little tolerance towards allylhalides, carboxylic acids, and alcohols. However, the catalyst could be used for the hydrosilylation of ketones and aldehydes. For the hydrosilylation of hex-5-en-2-one with Ph_2_SiH_2_, the selectivity was 9:1 alkene:ketone. If tetrahydrofuran THF as solvent was exchanged with dimethyl acetamide DMA, the selectivity could be further improved to 15:1.

Chakraborty et al. used bis(phosphinite) ligands (POCOP) to stabilize nickel-based pincer complexes [39]. The as-formed catalyst was highly efficient for the hydrosilylation of ketones and aldehydes. The cyanomethyl nickel complex showed TONs up to 82,000. In addition to its catalytic hydrosilylation activity, this complex system is capable of hydroboration of CO_2_ to methanol derivatives.

**Figure 16:**
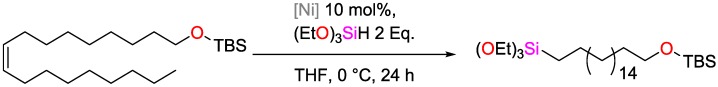
Hydrosilylation of TBS-protected oleyl alcohols.

The group of Buslov expanded the study of pincer-complexes to the development of novel catalysts based on nickel nanoparticles for the hydrosilylation of alkenes with tertiary silanes [40]. The hydrosilylation of tertiary silanes still is a challenging task for most hydrosilylation catalysts. One possible approach is an in-situ generated nickel alkoxide catalyst generated from stable and easy accessible reagents. In their study, the catalysts were prepared from nickel tetramethyl-ethylenediamine dichloride [(Ni-TMEDA)Cl_2_] and a metal alkoxide in THF as solvent. Experiments revealed Ni(O*^tert^*Bu)_2_·KCl as most active catalysts for the anti-Markovnikov hydrosilylation of dec-1-ene with trimethoxysilane TMS at yields of up to 88% using catalyst loads of 1 mol % with a TON of 850 and TOF of 1700 h^−1^. Additionally, this catalyst system showed high efficiency towards the tandem isomerization-hydrosilylation of internal alkenes with yields of up to 97% for the reaction of *n*-octene (both *cis* and *trans*). The hydrosilylation of hydrolyzed fatty acids protected with *^tert^*butylmethylsilyl TBS was investigated as well (Figure 16). Using a catalyst load of 10 mol %, protected oleyl alcohols were converted at 0 °C to yield saturated linear and ω-silylated protected alcohols with a yield of 45% and a terminal selectivity > 10:1. Hence, this catalyst is a potential candidate for the hydrosilylation of renewable resources.

Pappas and coworkers focused on the hydrosilylation of alkenes using an α-diimine nickel complex [41]. Stoichiometric studies indicated the presence of the catalyst as dimer [(2,6-*^iso^*Pr_2_-C_6_H_3_)NiH]_2_, while studies with deuterium labeling suggested the dissociation of the dimeric nickel complex which gives rise to the fast and reversible alkene insertion into the complex system. The catalyst showed high activity towards the hydrosilylation of oct-1-ene with TMS and derivatives up to 97% yield and 98% selectivity. Additionally, the reaction can be performed at r.t. and does not need pyrophoric activators. Along with its air stability and inexpensiveness, this complex is suitable for the commercial hydrosilylation of silanes and siloxanes.

**Figure 17:**
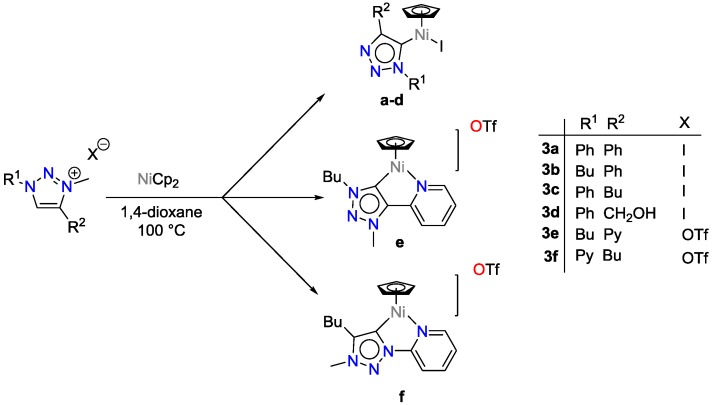
Synthesis of triazolylidene nickel complexes.

Wei et al. employed a number of nickel catalysts based on Ni^II^cp_2_, which were reacted with triazoles that carried alkyl, aryl pyridyl and methoxy groups as substituents (Figure 17) [42]. The choice of substituents played a major role for the selectivity towards the hydrosilylation reaction of carbonyl groups. Among the synthesized catalysts, one catalyst (namely 3e; Figure 17) was found to be the fastest and most efficient for the hydrosilylation of 4-methoxy benzaldehyde with TONs of up to 6000 and TOFs of 23,000 h^−1^. All catalysts showed high selectivity towards the hydrosilylation of aldehydes, even when large quantities of ketones were present. The catalysts can be easily prepared and are stable towards air and moisture; other functional groups are tolerated during the hydrosilylation. 

Steiman and Uyeda [43] investigated binuclear nickel catalysts. They found that the nickel-nickel bonds (along with redox-active ligands) constitute an excellent basis for catalytic activation. While these catalysts did not show any catalytic activity towards tertiary silanes, it may be argued that for activation binding to both nickel cores is required. The Ni^I^–Ni^I^ complex was tested for the hydrosilylation of alkynes, showing significantly higher yields (of up to 93%) for the reaction of Ph_2_SiH_2_ with alkynes than mononuclear nickel complexes.

**Figure 18:**
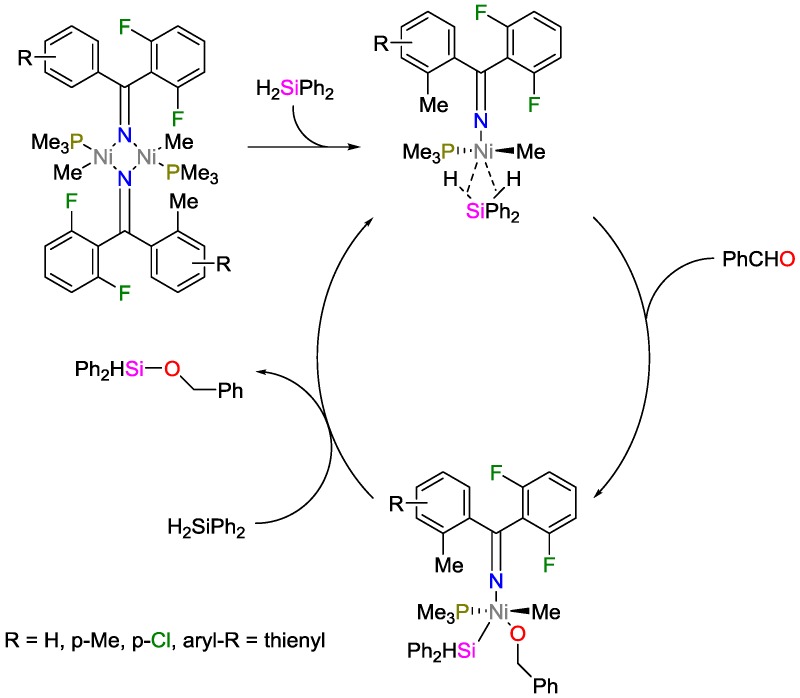
Mechanism of the catalysis by nickel-nickel-complex of the hydrosilylation of aldehydes.

Wang and coworkers [44] studied the reaction of NiMe_2_(PMe_3_)_3_ with fluoroarylimines that yields a binuclear imine/nitrogen-bridged nickel catalyst for hydrosilylation. These catalysts were tested with respect to catalytic activity towards the hydrosilylation of aldehydes. Among various silanes tested, Ph_2_SiH_2_ was found to be the best hydrogen source for the hydrosilylation of aldehydes, exhibiting conversions of up to 99%. A possible mechanism for the hydrosilylation of aldehydes catalyzed by binuclear catalysts was proposed (Figure 18). The authors suggested that the binuclear bond is first cleaved to form a mononuclear species containing the (μ^2^-Si–H)Ni^II^ motif. Preceded by cleaving one Si–H bond, the aldehyde is inserted. A second Ph_2_SiH_2_ molecule is engaged in the reductive elimination, while the silane-nickel species is recovered back into the cycle.

## 3. Non-Metal Catalysts for Hydrosilylation Reactions

Among the most commonly used non-metal catalysts are organic bases such as trialkylamines and Lewis acids, which will be discussed in the following paragraph. In the last few years, significant progress has been made in the development and utilization of novel Lewis acid catalysts for hydrosilylation reactions.

**Figure 19:**
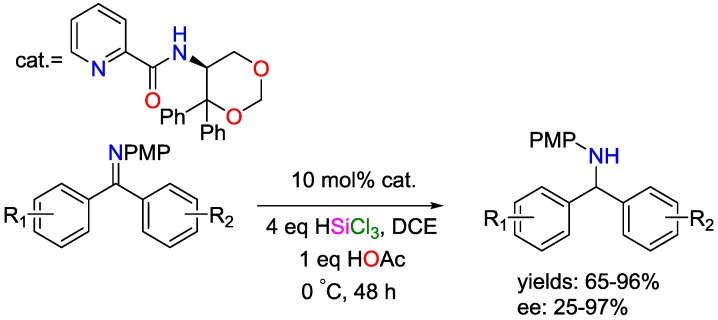
Asymmetric hydrosilylation of substituted benzophenone *N*-aryl imines.

Hydrosilylation reactions using equimolar amounts of organic bases were reported by Benkeser and coworkers [45]. They used tri-*n*-propylamine in the hydrosilylation reaction between benzylchloride and trichlorosilanes. For the reaction mechanism, the authors proposed the existence of trichlorosilyl anions, generated by interaction between the amines and silanes. The use of the silanes in combination with bases for the reduction of carbonyl compounds were discussed as well; here, the trichlorosilyl group can be easily cleaved under alkaline conditions [46]. Employing a *l*-serine derived (hence chiral) Lewis base, Zhang and coworkers reported a synthetic strategy for the asymmetric hydrosilylation of substituted benzophenone *N*-aryl imines with high yields and moderate to high stereoselectivity (Figure 19) [47].

Pike reported the addition of trichlorosilane to hydrocarbon olefins, catalyzed by tertiary amine and tertiary phosphine compounds (loads of 4–6 mol %) [48]. The trichlorosilane was added to terminal olefins; with non-terminal olefins such as pent-2-ene, branched-chain alkyltrichlorosilanes were obtained. On the contrary, platinum-catalyzed additions would yield straight-chained adducts in the latter case. Using the bases in a nitrile solvent system, trichlorosilane adds to hex-1-yne and phenylacetylene to yield the *trans*-adducts.

**Figure 20:**
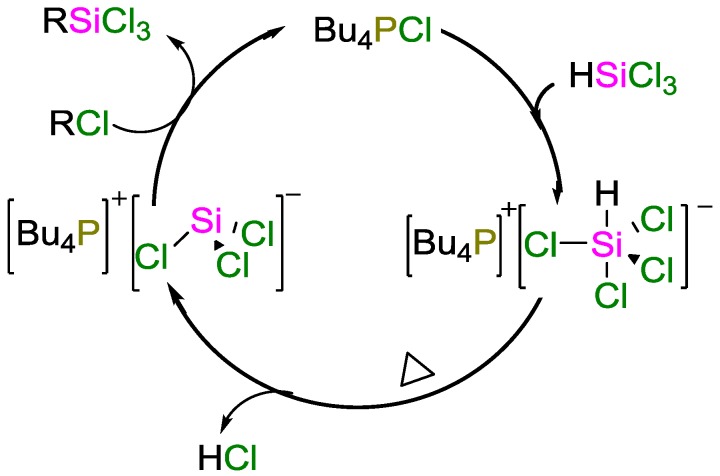
Possible hydrosilylation mechanism using a tetrabutyl phosphonium chloride catalyst.

Jung and coworkers reported a novel high-yielding hydrosilylation method using catalytic amounts of phosphonium salts [49]. The reaction proceeded even with non-activated chlorides such as methyldichlorosilane, which was not feasible under Benkeser reaction conditions, in which stoichiometric amounts of amines are required as hydrochloride scavangers. Using Bu_4_PCl, Jung et al. obtained the highest yields and conversions. Quantitative conversions of various benzyl chlorides with HSiCl_3_ were achieved at 130 °C within 4 h. Quarternary organoammonium chlorides were much less effective catalysts; non-activated alkyl chlorides together with ammonium catalyst failed to catalyze the product formation. With activated chlorides such as benzylchlorides, yields of the product were significantly lower (47% vs. 88% yield). A mechanism in which the phosphoric salts react with SiHCl_3_ to yield hydridotetrachlorosilane anions which eliminate HCl upon heating was proposed, according to which tetralkylphosphonium cation/trichlorosilylalanion pairs are formed (Figure 20). The as-formed intermediate can attack the alkyl chloride, achieve Si–C coupling, and regenerate the catalyst. Yoo and coworkers employed Bu_4_PCl for reactions wit HSiCl_3_ affording bis(chlorosilyl)methanes in moderate yields (up to 70%) [50].

**Figure 21:**
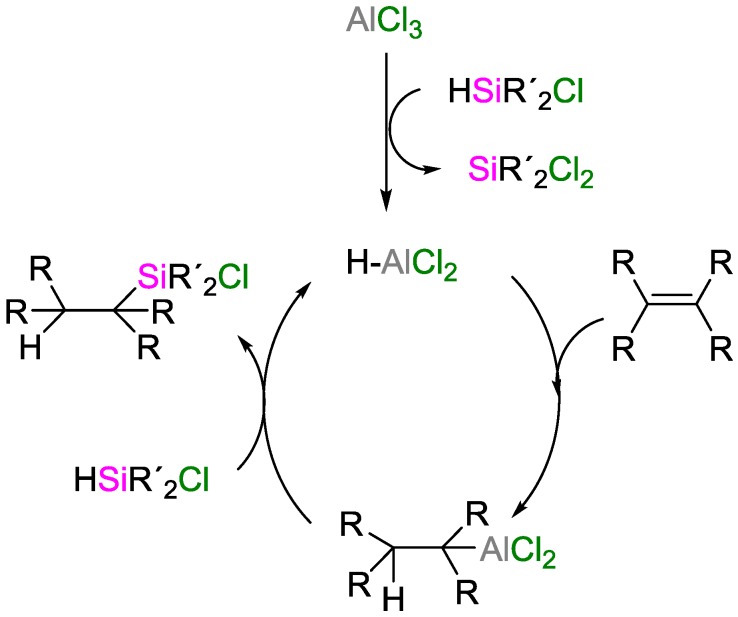
Mechanism of the hydrosilylation reaction catalyzed by AlCl_3_, proposed by Oertle and Wetter.

Oertle and Wetter reported the hydrosilylation of tetrasubstituted olefins with chlorinated hydrosilanes employing AlCl_3_ as catalyst [51]. They suggested a mechanism cycle initiated by the formation of hydroalane species from the reaction of AlCl_3_ with chlorinated hydrosilanes, followed by the hydroalumination of olefins and the transmetallation of alkyl alanes with hydrosilanes (Figure 21).

**Figure 22:**
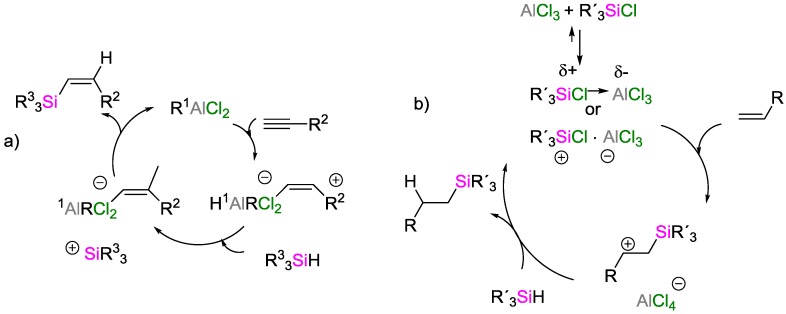
Mechanism of the hydrosilylation reaction catalyzed by aluminum compounds, proposed by: Yamamoto (**a**); and Jung (**b**).

A different mechanism for the hydrosilylation reaction catalyzed by aluminum compounds was proposed by the groups of Yamamoto [52] and Jung [53]. Their alternative mechanisms (Figure 22), unlike the originally proposed mechanism by Oertle and Wetter, differ in the nature of the key intermediates, for which they propose ionic species.

**Figure 23:**
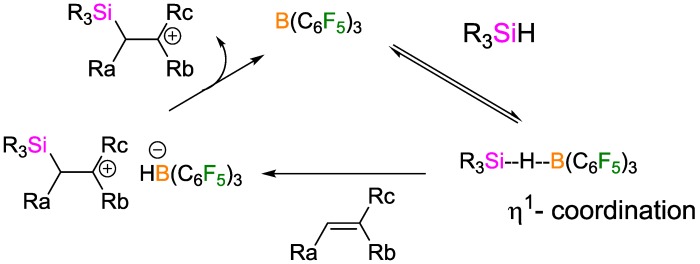
Possible mechanism of the hydrosilylation reaction catalyzed by B(C_6_F_5_)_3_.

Unlike transition metal catalysts, which catalyze the oxidative insertion of alkenes into the Si-H bonds, the Lewis acid non-metal catalysts activate the Si–H bonds via η^1^-coordination (Figure 23).

Chang and coworkers reported the metal-free hydrosilylation polymerization of dienes and with disilanes using the tris(pentafluorophenyl)borane catalyst [54]. Their method provided polymers with high polymerization degrees; various poly(carbosilane)s could be produced with a broad range of structures and properties. The reaction may be performed at r.t. with low catalyst loading of the commercially available catalyst, which can be easily removed from the product.

**Figure 24:**
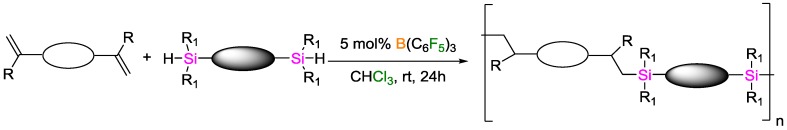
Borane-catalyzed hydrosilylation polymerization.

The borane catalyst B(C_6_F_5_)_3_ was successfully used in low loadings (5 mol %) for the homopolymerization of dienes such as styrenes [55]; however, such types of homopolymerizations could be successfully suppressed if silanes were additionally present in the reaction mixture (Figure 24). It was argued that the η^1^-coordination of silane to B(C_6_F_5_)_3_ suppresses the polymerization of alkenes, which is unique for B(C_6_F_5_)_3_: if, alternatively, AlCl_3_, is used as catalyst, large amounts of alkene homopolymerization by-products are formed. 2,2′-Dialkyl-substituted diene monomers are highly reactive towards polymerization, whereas the polymerization of less reactive non-substituted terminal dienes only yields oligomers of low molecular weight. The stabilization of the β-silylium cation intermediates is important to maintain high reactivity during the polymerization. The fact that tertiary carbocations are more stable than secondary ones (the latter formed in-situ from non-substituted dienes) is reflected in the difference of attainable polymerization degrees. 

**Figure 25:**
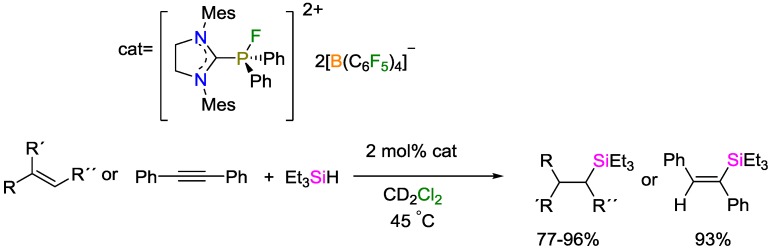
Hydrosilylation using Lewis acidic dicationic phosphonium salts.

Using organofluorophosphonium salts as catalyst in relatively low catalyst loading of 1.5 mol %, Stephan and coworkers reported high yields (above 89%) for the hydrosilylation of various olefins and alkynes [56]. The reactions were performed at r.t. Based on calculation studies, they proposed a mechanism of the hydrosilylation and the phosphonium activation of hydrosilane. The same research group reported the use of another type of Lewis acids as catalysts, namely dicationic imidazolium phosphonium salts (Figure 25), for the hydrosilylation of olefins [57]. This type of catalysts does not require strongly electron-withdrawing substituents at the phosphonium center, paving the way to broad structure variations. Using 2 mol % of this catalyst, various olefins and alkynes could be hydrosilylated in high yields.

**Figure 26:**
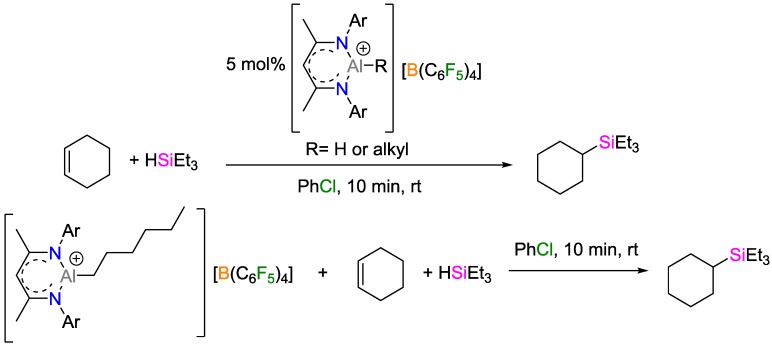
Hydrosilylation reaction of cyclohexene catalyzed by a cationic aluminum complex.

Nikonov and coworkers reported the hydrosilylation of olefins catalyzed by the cationic [CH{C(Me)N(2,6-*^iso^*Pr_2_C_6_H_3_)}_2_–AlH]^+^ aluminum complex [58]. Quantitative hydrosilylation of terminal alkenes and cyclohexene at r.t. within 10 min using 1 mol % of catalyst was observed. Despite the facile olefin insertion into the Al–H bond (as proposed by Oertle and Wetter; see above), the catalysis does not proceed via the insertion mechanism. Since no reaction of the alkene-inserted product with the silane could be observed, a mechanism that proceeds via Lewis acid activation was proposed; the activation of silanes in the same manner as by boranes as previously reported by Piers and Oestreich was argued [59,60,61,62,63,64]. The [Et_3_Si-H∙∙∙Al(C_6_F_5_)_3_] complex (Figure 26) has been reported by Chen and coworkers [65] who have suggested a detailed mechanism of hydrosilylation. The initial activation of the silane is followed by the alkene attack of the silylium ion, yielding a carbocation intermediate that undergoes hydride abstraction from [CH{C(Me)N(2,6-*^iso^*Pr_2_C_6_H_3_)}_2_-AlR]^+^ to yield the alkylsilane. The drawback of the applicability of this catalyst is the limitation of solvents as reaction media: in many nonpolar solvents, the catalyst precipitates, while, on the other hand, it decomposes in polar solvents such as dichloromethane and tetrahydrofuran. Stability of the catalysts for at least 1 d was verified in chlorobenzene. 

**Figure 27:**
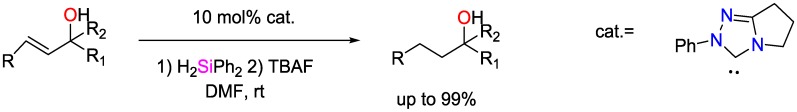
Reduction of 3-aryl allyl alcohols.

*N*-heterocyclic carbenes NHCs have as well been demonstrated to catalyze hydrosilylation reactions. They have been employed by Lacôte and coworkers in the chemoselective reduction of olefins and alkynes, which contained α-hydroxy substituent, through silyl ether formation (Figure 27) [66]. They demonstrated that not only NHCs, but also cyclic alkylaminocarbene CAACs can catalyze these reactions. The reactions are limited to aryl-substituted reactants.

**Figure 28:**
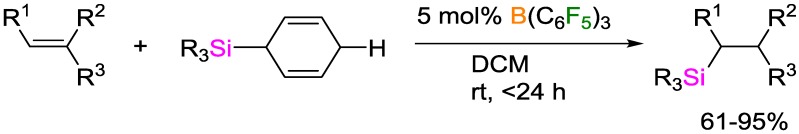
Reaction path for the hydrosilylation of olefins with in-situ generated Me_3_SiH or Me_2_SiH_2_.

Simonneau and Oestrich [67] reported the use of 3-silylated cyclohexadi-1,4-enes as precursors for the in-situ generation of the gaseous hydrosilanes Me_3_SiH and Me_2_SiH (Figure 28). They used 5 mol % of the Lewis acid catalyst B(C_6_F_5_)_3_ with various alkene and styrene derivatives at r.t. In a subsequent study, they performed a systematic study of various Lewis acids that could be used for this particular reaction [68].

**Figure 29:**
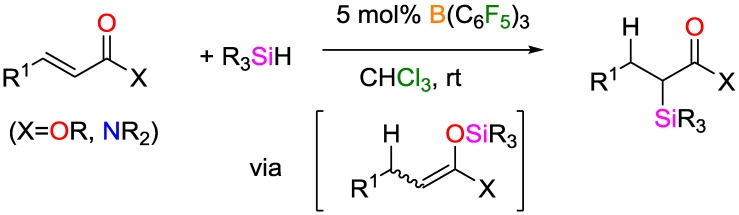
Selective hydrosilylation of α,β-unsaturated carbonyls.

A novel method for obtaining α-silyl carbonyl products using B(C_6_F_5_)_3_ as catalyst was reported by Kim and Chang [69]. Unlike previously reported hydrosilylations of unsaturated polar bonds (such as carbonyls, imines, and nitriles [70,71,72]), the authors demonstrated the feasibility of the chemoselective α-silylation of conjugated esters and amides, in which the carbonyl group was left intact (Figure 29). According to preliminary mechanistic studies, the reaction proceeds in two steps, namely rapid 1,4-hydrosilylation of conjugated carbonyls and slow silyl group migration of a silyl ether intermediate. 

Oestreich and coworkers reported the highly enantioselective hydrosilylation of acetophenone derivatives (up to 99% *ee*) [73], employing an axially chiral cyclic borane that contained only one C_6_F_5_ group at the boron atom. It was demonstrated that the borane catalyst promoted the hydrosilylation without a need for an additional Lewis base.

## 4. Non-Thermal Stimuli for Hydrosilylation Reactions

Intense research has been dedicated to the development of catalysts that can be switched by external triggers and demonstrate different activity and selectivity [74,75]. Making a hydrosilylation reaction switchable represents a huge challenge as it commonly proceeds immediately at high speed in the presence of most catalysts at r.t. For some industrial applications, however, it is required to have a curable formulation composed of catalyst and crosslinking components that are shelf-stable (storable) for multiple months. Such specialized and growing demands for polysiloxane products were the main drive to develop hydrosilylation systems that can be started upon the application of a certain external trigger. Heat has been the most commonly used trigger in industrial applications so far. Various examples of thermally activated hydrosilylation have already been covered in the review by Troegel and Stohrer [6], where various inhibitors of platinum catalysts, platinum catalysts containing inhibiting ligands and encapsulated catalysts were described. In this review, advances in the field of non-thermally triggered hydrosilylation reactions will be described.

### 4.1. Light-Initiated Hydrosilylation Reactions

Light/irradiation presents a very attractive stimulus as it allows precise spatiotemporal control, which makes it a highly favorable trigger for industrial applications. Initial approaches towards photo-triggerable hydrosilylation reactions involved the photo-activation of various metal hexacarbonlys, namely Cr(CO)_6_, Mo(CO)_6_, Fe(CO)_6_ and Ni(CO)_6_. However, these catalysts tend to agglomerate, are often non-selective, and only achieve mediocre degrees of conversions [76]. Most commonly used and well-studied photo-initiatable catalysts are platinum-based ones such as a pioneer example, namely the platinum/oxalate complex Pt(C_2_O_4_)L_2_, which can be photochemically reduced [77,78], yielding a reactive platinum center (commonly bound to triaryl or trialkyl phosphines).

Platinum complexes of triazenes are both heat-sensitive and photolabile. Wokaun and coworkers demonstrated that tetrakis(1-phenyl-3-hexyl-triazenido)platinum^IV^ can be activated by a XeCl excimer laser at 308 nm to subsequently catalyze the reaction between a divinyl-disiloxane and a dihydro disiloxane [79]. In addition to the targeted (AB)_n_ polymer chains, by-products such as cyclic compounds, isomers, and hydrogenated vinyl groups were observed as well.

**Figure 30:**
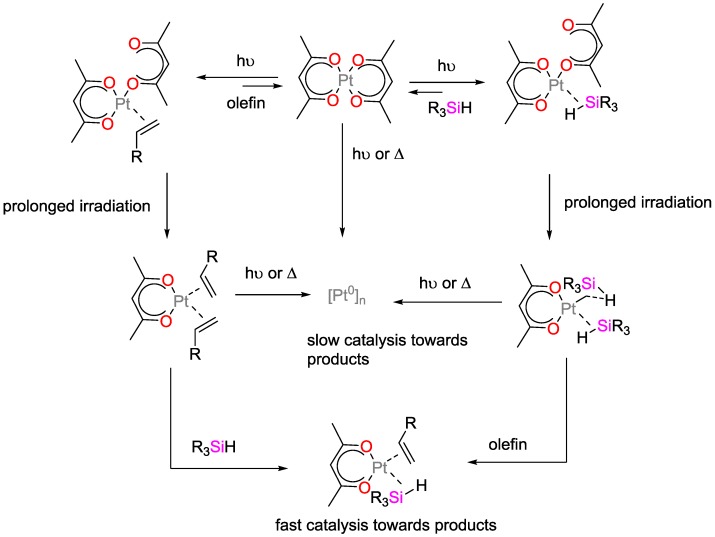
Proposed mechanism for the photo-initiated generation of a highly active platinum catalyst from Pt(acac)_2_.

Lewis and Salvi studied Pt^II^-bis(β-diketonates) as photo-activatable hydrosilylation catalysts (Figure 30) [80]. They found that irradiation initially yields a highly active homogenous catalyst, which is thermally converted to a less active heterogeneous catalyst. They demonstrated that the formation of the active catalyst requires the presence of one of the reactants during a brief period of irradiation. It was suggested that the primary photoproduct was not the catalytically active species, but that a secondary photochemical reaction resulted in the loss of one of the two β-diketonate ligands, which finally yields the active catalyst. If the irradiation was terminated after the conversion of, e.g., Pt(acac)_2_ to the primary photoproduct, and the complementary reactant was added only at that stage, hydrosilylation did not occur. In the presence of excess hydrosilane or olefin, however, the activated catalyst maintained its activity for several hours at r.t. and was slowly converted to colloidal Pt. 

The platinum catalyst (η^5^-C_5_H_4_CH_3_)Pt^IV^(CH_3_)_3_ was studied by Lees and Jakubek [81], who characterized its photochemistry in detailed fashion. Dissolved in methylcyclohexane and pentane at 293 K, the complex showed high quantum efficiencies at 311 and 366 nm ranging from 0.34 to 0.41; upon incorporation of Et_3_SiH, it even exhibited quantum efficiencies in the range from 0.79–0.85. The compounds were effective photo-initiators for the hydrosilylation reactions involving vinyl/hydride silicone mixtures for catalyst loadings higher than 520 ppm. 

Boardman investigated (η^5^-cp)Pt^IV^R_3_ complexes as photoactive hydrosilylation catalysts; they argued that the active catalyst species were platinum colloids since the catalyst could be poisoned by elementary mercury but was not inhibited by dibenzo [a,e] cyclooctatetraene [82].

**Figure 31:**
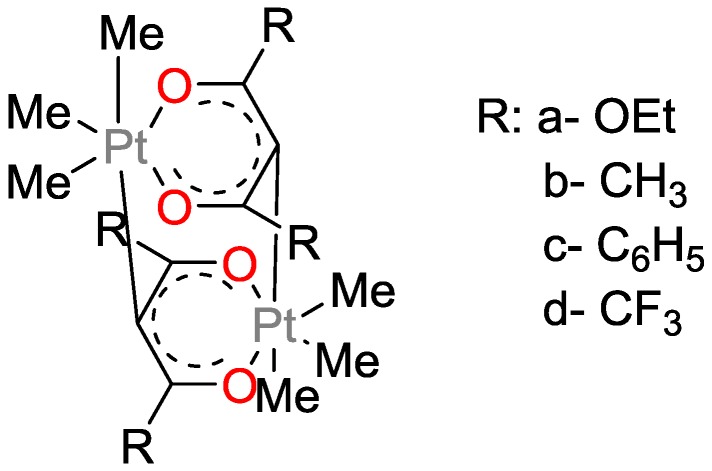
Dimeric structures of β-dicarbonyl-Pt^IV^R_3_ complexes employed by Fouassier and coworkers.

Fouassier and coworkers employed various β-dicarbonyl-Pt^IV^R_3_ complexes (Figure 31) as useful photoinitators for hydrosilylation reaction of silicone polymers containing Si–H/Si–vinyl and Si–H/Si–epoxide moieties [83]. During photolysis of the initiator, colloidal platinum was generated. The reactivity of the photoninitiators was enhanced by the presence of electron donors at the carbonyl C-atom of the β-dicarbonyl ligand. Thieulux, Meille and coworkers reported that colloidal suspensions of monodisperse platinum nanoparticles of 2 nm were able to catalyze the hydrosilylation of oct-1-ene with poly(methylhydroxysilane) [84]. The nanoparticles were found to be as efficient as Karstedt’s complex, showing that colloid formation from homogenous species during hydrosilylation is not necessarily a deactivation pathway.

Fry and Neckers employed Pt(acac)_2_ for the photoactivated hydrosilylation polymerization of vinyldimethylsilane [85]. They observed a significant degree of heterogeneous platinum catalysis in the mechanism of the polymerization: The polymers doubled molecular weights during ageing due to the reaction of telechelic residual vinyl and hydride groups; this reaction was apparently catalyzed by platinum colloids formed after photolysis. 

Vekki and Skvortsov studied the hydrosilylation of vinylsiloxanes in the presence of thermo- and photo-activatable Pt^II^-phosphine complexes [86]. Using thermal activation, the reaction rate decreased in the order: *cis*-[Pt(PMe_2_Ph)_2_Cl_2_)] > *cis*-[Pt(PPh_3_)_2_Cl_2_)] > *cis*-[Pt(PBu_3_)_2_Cl_2_)]. The induction period shortened in the following ligand order: Ph_3_P < PhMe_2_P < Bu_3_P. The influence of the phosphine ligands on the efficacy of the photoactivated siloxane notably differed from that in the dark, and the induction period shortened in the order: Ph_3_P > Bu_3_P >> PhMe_2_P. Oxygen accelerated the hydrosilylation catalyzed by photoactivated Pt^II^-phosphine complexes, whereas the reaction rate decreased in argon atmosphere. This observation was explained by oxidation of one of the phosphine ligands, which disabled it from coordinating to the platinum ion and helped forming the active species faster. 

The hydrosilylation of low-molecular siloxanes in the presence of various photoactivated sulfoxide-Pt^II^ complexes was investigated by the same research group [87]. Additional photosensitive centers, such as oxalate ligands, yielded more efficient photo-initiatable systems. High selectivity and conversions could be achieved with short irradiation times. 

Neckers and coworkers employed Pt(acac)_2_ for the crosslinking of oligo [(methylsilylene)-methylene] OMSM [88]. The formation of crosslinked polysilanes from liquid mixtures could be completed within a minute (solidification of the reaction mixture).

Marchi and coworkers reported hydrosilylation reactions using (η^5^-C_5_H_4_CH_3_)Pt^IV^(CH_3_)_3_ in the presence of various amounts of the 2-chlorothioxanthen-9-one CTX sensitizer. Activation of this system could be accomplished by visible light [89]. Using 2.4 mol % of the platinum catalyst, minimum dosage of CTX had to be at least 4000 ppm to reach reasonable silane conversion upon irradiation with wavelengths of 380–515 nm. Even with loadings of photosensitizer that high, the silane conversion was lower in comparison to the UV-activated hydrosilylization (85%). 

**Figure 32:**
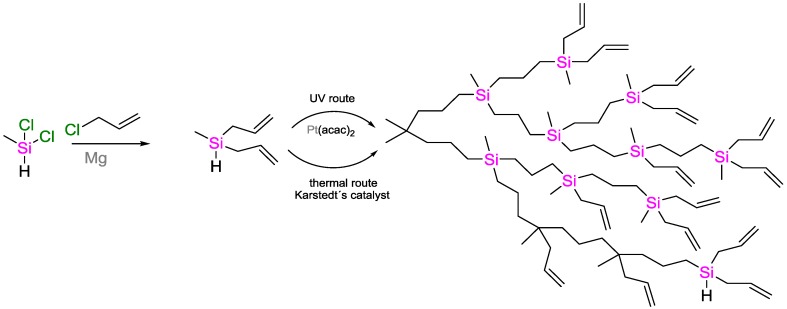
Synthesis route of hyperbranched poly(carbosilane)s via UV-activated or thermally activated polymerization.

Huang and coworkers performed the synthesis of hyperbranched poly(carbosilane)s (Figure 32) [90]. The UV-activated hydrosilylation using Pt^II^(acac)_2_ catalyst was found to be more efficient (reaction finished in 40 min) than the classical thermal activation using Karstedt’s catalyst (5–6 h duration of the reaction). Since the reactions were performed in small scale in toluene only (1.26 g of the reactant methyldiallylsilane), it is hard to predict if the effect could be reproduced on large scale relevant for industrial applications.

Marchi and coworkers demonstrated in how far (in terms of energy and time saving) photo-activation is the favorable way for (crosslinking) hydrosilylation catalysis employing Pt^II^(acac)_2_ as catalyst [91]. They used the catalyst for photo- and thermal activation and monitored the silane conversion at various temperatures. UV activation was applied at r.t. and the reaction was completed within 5 min (conversion of 98%), whereas, after 150 min of thermal curing at 150 °C, the reaction showed a lower conversion of 82%. The increase of the glass-transition temperature *T*_g_ revealed that the UV route led to more densely crosslinked materials. The same research group employed Pt(acac)_2_ catalysts for the curing of various silicone composites such as PDMS composites by UV-triggered hydrosilylation [92]. In the absence of any inorganic fillers, they performed the UV-triggered curing of 4 cm thick samples of silicone polymers based on, e.g., vinyldimethylsiloxane-terminated PDMS [93]. They reported the UV-induced curing of samples of up to 2 cm of silicone composites containing inorganic fillers, obtained via hydrosilylation and in-situ formed inorganic particles [94].

Sangermano and coworkers demonstrated why the curing of comparably thick samples of up to 4 cm is possible with UV-activated Pt(acac)_2_ [95]. They argued that a (thermal) polymerization front may be generated as the polymerization reaction is exothermic, in which the UV-generated homogenous catalyst is converted to a heterogeneous colloidal catalyst. The UV-induced frontal polymerization mechanism may also be used to explain the dark curing process observed in the UV-activated hydrosilylation reaction. They demonstrated that according to the same mechanisms samples of even up to 5 cm could be cured using the photoactivated (η^5^-C_5_H_4_CH_3_)Pt^IV^(CH_3_)_3_ complex [96].

Neckers and coworkers demonstrated the use of various bis(β-diketonato)Pt^II^ complexes for the hydrosilylation cross-linking reaction of oligo [methyl-silylene)-methylene] with tetravinylsilane. The platinum catalysts contained ligands such as benzylacetonate, trifluoroacetylacetonate, and benzoyltrifluoroacetonate and could be photoactivated by visible light [97].

**Figure 33:**
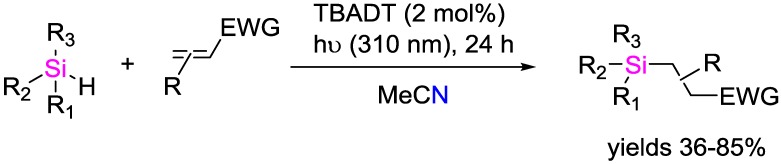
TBADT-photocatalyzed hydrosilylation of electron-poor olefins.

The same research group reported the photocatalytic generation of silyl radicals from trisubstituted silanes by tetrabutylammonium decatungstate TBADT (Figure 33) [98]. Hence, the photocatalytic hydrosilylation of electron-poor alkenes could be performed. The reactions could be triggered by light of 350 nm wavelength; in some cases (e.g., addition to maleates), the reaction also proceeded efficiently under sunlight as well as under flow conditions. 

**Figure 34:**
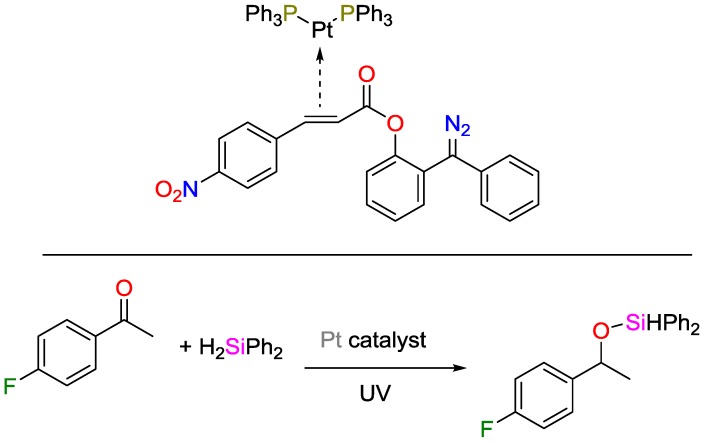
Catalytic hydrosilylation of *p*-fluoro-acetophenone using novel light-sensitive platinum-based catalysts.

UV-triggered catalysis, even though intensively explored for decades, is not yet fully understood, and only a few catalysts relying on it are described in detailed fashion in the literature [99]. Ruhland and coworkers developed a novel platinum-based catalyst for hydrosilylation that contained the photo-active moiety in the outer ligand sphere Figure 34 [100]. Upon irradiation at 300 nm, this moiety could react with the inner ligand sphere, yielding a free coordination site at the metal center. They developed three novel ligands based on azo compounds and showed that the as-derived catalysts can be triggered by UV stimuli. 

**Figure 35:**
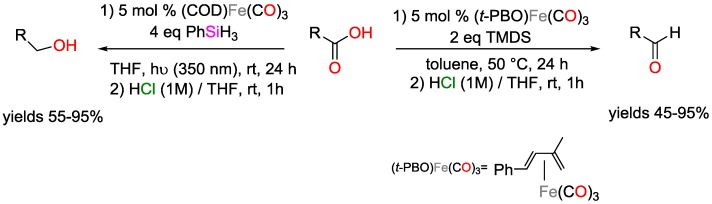
Selective reduction of carboxylic acids to either alcohols or aldehydes using iron-catalyzed hydrosilylations.

Stimuli-induced iron-catalyzed hydrosilylation of carboxylic acids was reported by Darcel and coworkers (Figure 35) [101]. (COD)Fe(CO)_3_ (COD: cyclooctadiene) efficiently catalyzed the reduction of carboxylic acids to alcohols at r.t. under UV irradiation (350 nm) if phenylsilanes were present as silane source. On the other hand, the reduction of the carboxylic acids to aldehydes could selectively be obtained using 1,1,3,3-tetramethyldisloxane TMDS as silane source and (*t*-PBO)Fe(CO)_3_·(*t*-PBO: *trans*-4-phenyl-but-3-en-2-one) as catalyst under thermal activation. If TMDS was used with (COD)Fe(CO)_3_ in THF under UV irradiation, the major products were aldehydes and not alcohols, revealing that the silane source has significant role in determining the reduction pathway. 

A reversibly switchable hydrosilylation process (that can be turned ON and OFF by stimuli) was reported by Grzybowski and coworkers [102] on the example of the hydrosilylation reaction of 4-methoxybenzaldehyde with diphenylsilane in toluene. As catalyst, photo-switchable gold nanoparticles were used. The gold nanoparticle surfaces were covered with photoresponsive azobenzene-thiol ligands and a surfactant. Non-aggregated nanoparticles have a large surface area and may catalyze the reaction. Upon irradiation at 365 nm, azobenzene undergoes *trans-cis* isomerization, enhances its electric dipole, and causes the particles to aggregate, which switches off the reaction. It can be reactivated upon exposure to the visible light. The drawback of this system is that the switchability is based on aggregation mode of the catalyst. That means it only works in certain solvents, making it unattractive for industrial applications or any larger scale initiations of hydrosilylation.

### 4.2. Solvent as a Trigger to Influence the Stereochemical Configuration of Hydrosilylated Products

**Figure 36:**
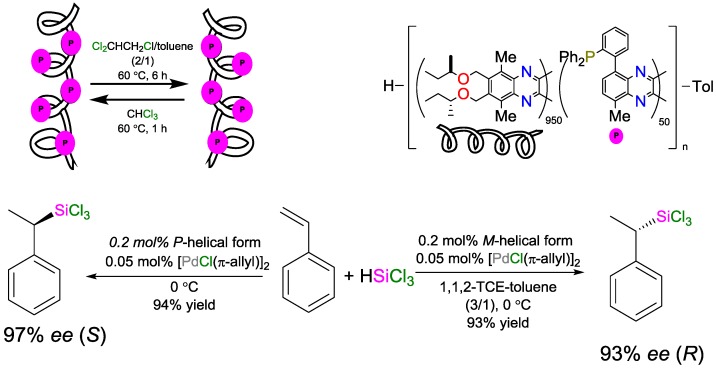
Asymmetric hydrosilylation using a polymeric ligand with switchable chirality in a platinum-catalyzed hydrosilylation. The polymeric ligand adjusts its helicity to the solvent.

Suginome and coworkers reported the asymmetric hydrosilylation of styrene [103]. They employed a polymeric ligand based on poly(quinoxaline phosphine) as catalyst in the platinum-catalyzed hydrosilylation reaction; depending on the solvent used as reaction medium, the polymer formed different secondary structures (different helicity), which translated into the different enantiomers of the product. Using very low catalyst loadings, silylated products were obtained in very high yields and enantioselectivities (Figure 36); the catalysts could be used in eight consecutive catalytic cycles without any loss of activity or selectivity.

The same group reported another copolymer based on poly(quinoxaline-2,3-diyls) and employed it in a switchable asymmetric hydrosilylation reaction [104]. However, unlike the previous copolymer which was synthesized from a chiral monomer, the novel copolymer was prepared from a monomer derived from (*R*)-octan-2-ol (23% *ee*) and a monomer bearing a PPh_2_ group that adopted a single-handed helical structure. The helical structure of the ligand could be modified as the previous by changing the solvents. Asymmetric hydrosilylation was demonstrated on the platinum-catalyzed example of the reaction of β-methylstyrene with trichlorosilane using this polymeric ligand; excellent yields and enatiomeric selectivity (93% *ee* for the *S* product and 94% *ee* for the *R* product) were obtained.

### 4.3. Microwave-Initiated Hydrosilylation Reactions

While microwave irradiation transfers heat to any materials with pronouncedly separated (partial) charges such as metals, ions and dipoles, it is hard to discriminate between “regular” thermal initiation and the so-called non-thermal microwave effects. Ozin and coworkers demonstrated that microwave-assisted heating had no evident acceleration effect on the hydrosilylation rate relative to conventional thermal heating (hydrosilylation kinetics of hydride-capped silicon nanocrystals with dec-1-ene) [105].

Boukherroub and coworkers reported the functionalization of hydrogen-terminated porous silicon surfaces with functional alk-1-enes under microwave irradiation [106]. Organic monolayers covalently attached to surface by Si–C bonds were argued to originate from microwaves as a source of energy that accelerated the hydrosilylation reaction and yielded a higher surface coverage compared to conventional heating; performance of the reaction at 180 °C in an oil bath hardly yielded any chemical grafting on the particles’ surfaces. Under microwave irradiation for 30 min at 170 °C, the reaction efficiency was reported to be 38%, but was not further improved upon increasing the temperature. Porter and coworkers as well reported the alkyl-functionalization of porous silicon via multimode-microwave assisted hydrosilylation [107]. 

### 4.4. Sonication-Triggered Hydrosilylation Reactions

Various activation methods have been developed for the hydrosilylation reaction on hydrogen-terminated surfaces (e.g., silicon surfaces in the wafer industry) including the use of radical initiators such as diacyl peroxides [108], catalysts such as H_2_PtCl_6_ [109], as well as electrochemical [110], photochemical [111], thermal [112,113], and microwave-assisted activation methods. 

**Figure 37:**
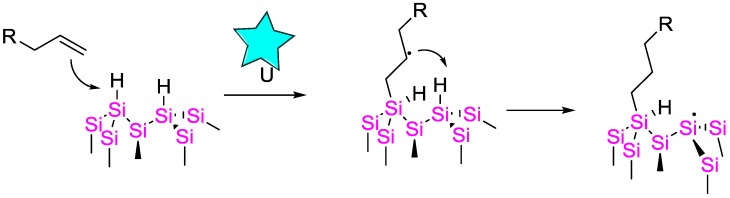
Proposed mechanism of the sonochemical activation of hydrogen-terminated silicon surfaces for hydrosilylation reactions with terminal alkenes.

A sonochemical activation method was reported by Zhong and Bernasek [114], who used an ultrasonic bath at r.t. for the performance of a hydrosilylation reaction; catalyst and/or radical initiator did not need to be added. They achieved high degrees of functionalization with various alkenes. The surfaces could be selectively functionalized with bifunctional alkenes such as undecenol, undecylenic acid and even heat/UV sensitive alkenes bearing an activated leaving group such as *N*-succinimidyl undecylenate. The authors suggested that acoustic cavitation activates the hydrogen-terminated silicon surfaces for hydrosilylation selectively with the vinyl group, forming a self-assembled monolayer SAM terminated by a second group available for further chemical modifications. The proposed mechanism (Figure 37) suggests that the radical formed by sonication reacts with the alkene forming an alkyl radical intermediate. The newly formed radical intermediate then abstracts hydrogen from an adjacent Si atom, forming another surface Si radical available for further reaction.

Bernasek and coworkers investigated the substituent effects in bifunctional styrenes on the SAM formation on hydrogen-terminated silicon surfaces [115]. They studied methylstyrene, styrene, chlorostyrene, cyanostyrene, and trifluoromethylstyrene. Except for trifluoromethylstyrene, all styrenes were bound to the surface only through the alkene moiety.

### 4.5. Electrochemically Initiated Hydrosilylation Reactions

**Figure 38:**
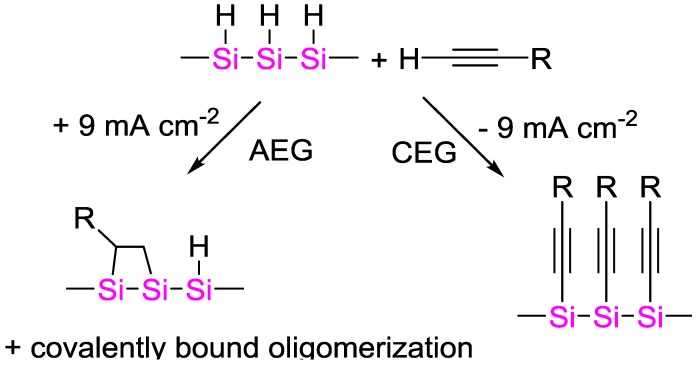
Cathodic and anodic electrochemical grafting.

Buriak and coworkers demonstrated that terminal alkynes can be electrochemically grafted to porous silicon surfaces with either positive or negative bias [110]. Via cathodic electrografting CEG, alkynes were directly attached to the surface, whereas with anodic electrografting AEG an alkyl surface was generated. The authors proposed that CEG proceeded via a silyl anion intermediate formed by the reduction of surficial Si–H bonds. The subsequent in-situ generation of a carbanion from deprotonation of weakly acidic alkyne leads to a nucleophilic Si–Si bond attack. It is likely that surface-initiated cationic hydrosilylation mechanism is responsible for Si–C bond formation in AEG reactions (Figure 38).

## 5. Industrial Applications

As demonstrated in the previous paragraphs, numerous catalysts are currently present with which hydrosilylation reactions can be successfully performed. For the implementation of hydrosilylations into industrial processes, catalysts have to be low-priced and show high selectivity, efficiency and stability. However, highly-functional products may be calculated on an elevated price scale [6]. Hence, apart from high efficient/low-cost catalysts possibly paving the way for industrial exploitation, recent advances in hydrosilylation reactions of functional products will be presented. 

### 5.1. High Efficient/Low-Cost Hydrosilylation (Solvent-Free Conditions)

The vast majority of hydrosilylation processes in industrial manufacturing are still performed using platinum-based catalysts. Platinum itself is one of the most expensive metals and, correspondingly, many hydrosilylation processes suffer from the disadvantage that the platinum catalyst cannot be recovered. It is desirable to employ different transition metals which could replace expensive platinum. The replacement of expensive, sensitive and hazardous chemicals such as reducing agents and solvents with stable and easy-to-handle chemicals will further decrease the process costs.

**Figure 39:**
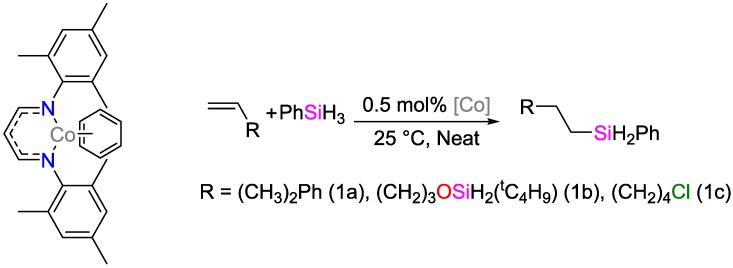
Hydrosilylation of alkenes under solvent-free conditions.

Chen et al. reported a cobalt-based catalyst for the anti-Markovnikov alkene hydrosilylation under solvent-free conditions at low temperatures and low catalyst loadings (Figure 39) [116]. Additionally, the catalysts can be synthesized in-situ from commercially available, low-cost precursors under ambient conditions giving high yields/selectivity for functionalized alkenes (namely 1a = 89%/>98%, 1b = 94%/98%, 1c = 89%/98%). 

**Figure 40:**
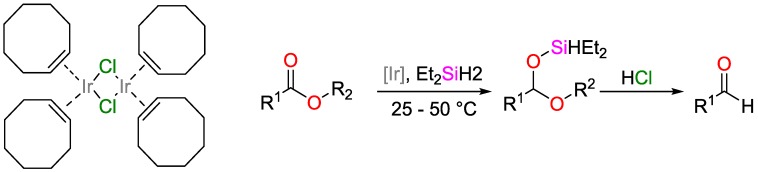
Hydrosilylation of esters followed by acidic reduction to yield aldehydes.

The reduction of esters to aldehydes is often performed with nucleophilic hydride agents such as di-*^iso^*butylaluminium hydride DIBALH. However, these agents are air and moisture sensitive, toxic, and tend to reduce the aldehyde directly towards the alcohol [117]. To prevent over-reducing and provide easier process conditions, Cheng et al. proposed a simple and highly efficient method of reducing esters to aldehydes using hydrosilylation (Figure 40). The iridium-based catalyst [{Ir(COE)_2_Cl}_2_] (coe = cyclooctene) is commercially available, offers high conversion (99%) at low catalysts loading (0.1 mol %), and shows good compatibility towards functional groups such as aryl and alkyl halides, alkenes and alkynes, tertiary amines and nitrides as well as hydroxyl and sulfonyl groups [118].

Wekesa et al. reported the use of a bis(arylimino)acenaphthene BIAN complex based on iron that could be used for the solvent-free hydrosilylation of ketones and aldehydes with up to a 99% yield [119]. The hydrosilylation of benzaldehyde with mono- and diphenylsilane was completed within 1 h. The complex shows high catalytic activity towards various other ketones and aldehydes with high yields ranging from 85–98%.

**Figure 41:**
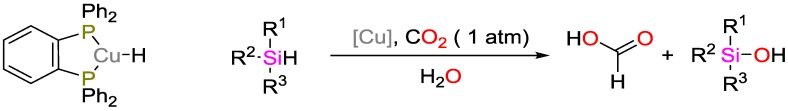
Hydrosilylation of CO_2_ yielding silanols and formic acid.

The use of renewable feedstocks is a generally favorable pathway towards greener standards in industrial applications. Motokura et al. proposed to use a copper catalyst for the hydrosilylation of CO_2_ to generate silanols and formic acid in a one-pot reaction (Figure 41) [120]. The high yield of up to 95% and TONs of 8100, however, are counterbalanced by the high prices of hydrosilanes. Zhang et al. also proposed to use a NHC-copper alkoxide complex for the hydrosilylation of CO_2_ with triethoxysilyl compounds [121]. Although the yield dropped to 75% and the TON to 7500, the whole reaction benefited from the fact that it could be carried out at 60 °C under solvent-free conditions instead of using high temperature such as 100 °C in solvents.

**Figure 42:**
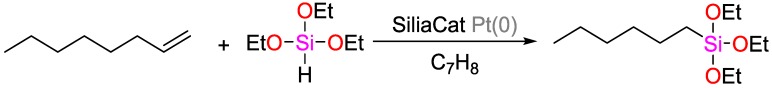
Immobilized platinum nanoparticles for the hydrosilylation of oct-1-ene.

It is still a big challenge for other complex systems to compete with the high catalytic activity of Speier’s and Karstedts’s catalysts. However, a possible route to lower process costs is to minimize the consumption of catalyst. Cririminna et al. reported the use of platinum nanoparticles incorporated into a silicate porous matrix for the hydrosilylation of long-chained alkenes (Figure 42) [122]. The benefit of the system is the minimized leaching of catalyst during reaction, preventing loss of the catalyst and contamination of the product. The catalyst can be restored by sonication in CH_2_Cl_2_ and be reused at least three times. Scale-up experiments from 2 to 100 mmol substrate indicate no loss of selectivity or conversion degree at higher amounts. [123]

**Figure 43:**
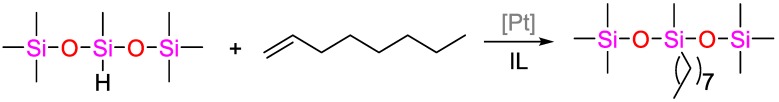
Hydrosilylation of 1,1,1,3,5,5,5-heptatrimethylsiloxane with oct-1-ene in ILs using the platinum-based Karstedt’s catalyst.

Other approaches to improve the durability of platinum-based catalysts and to further optimize the hydrosilylation process is the use of ionic liquids ILs as well as microreactors. Microreactors enable the performance of continuous chemical reaction process in tubes with small diameter, which results in higher yields due to a high surface:volume ratio and therefore increased mass transfer [124]. Studies performed by Kukawa et al. indicate higher yields (98%) for the hydrosilylation of 1,1,1,3,5,5,5-heptatrimethylsiloxane with oct-1-ene in ILs when using a continuous flow reactor (Figure 43). This set-up enables the continuous substrate addition and continuous product separation, which is a great advantage considering the potential for industrial application. Additionally, no catalyst was found in the product after the reaction allowing a reuse of the IL and catalysts [125].

**Figure 44:**
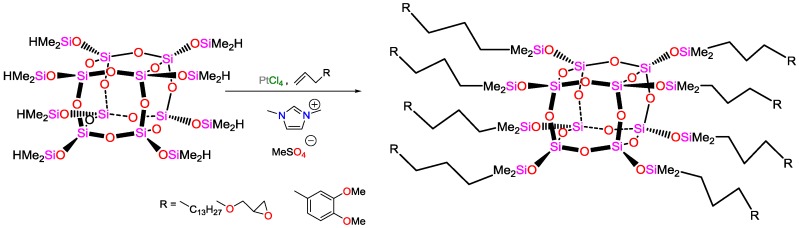
Hydrosilylation of octakis(hydridodimethylsiloxy)octasilsesquioxane (HMeSiO)_8_ [SiO_1.5_]_8_.

Maciejewski and coworkers proposed an easy and highly efficient hydrosilylation for the synthesis of organofunctional polyhedral oligomeric silsesquioxanes oPOSS (Figure 44) [126]. As catalyst, PtCl_4_ immobilized in 1,2,3-trimethylimidazolium methylsulphate was used. The product is insoluble in the IL, hence product isolation is fast and easy, and the catalyst can be reused for further reactions without loss of activity. 

### 5.2. Recent Advances in Functional Materials Using Hydrosilylation

Hydrosilylation is a versatile reaction delivering materials for fields of application comprising anti-fouling coatings and coatings for medic(in)al applications [127,128], as well as functionalized surfaces and packaging materials for electronic applications [129,130], organic, and hybrid-organic/inorganic semiconductors [131,132].

#### 5.2.1. Coatings

**Figure 45:**
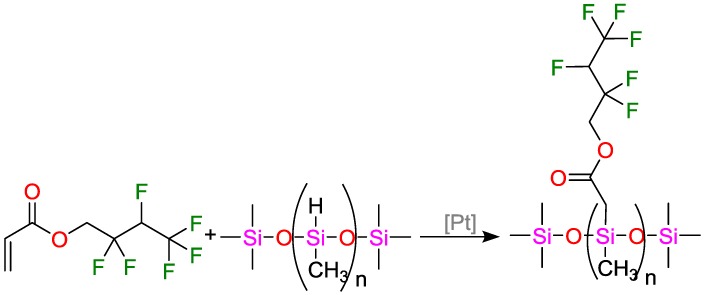
Grafting of PMHS with hexa-fluorobutyl acrylate using a Karstedt’s catalyst.

Liu et al. prepared an anti-graffiti film by incorporating a poly(methyl hydro siloxane) PMHS polymer grafted with hexa-fluorobutyl acrylate into polyurethane (Figure 45) [133]. The free surface energy was reduced from 30.7 (polyurethane) to 21.4 mN·m^−1^ (anti-graffiti-polyurethane). Due to the lower free surface energies, the wetting capabilities were correspondingly deteriorated. Cleaning tests revealed that anti-graffiti-coated areas could be completely cleaned using water and isopropanol. 

**Figure 46:**
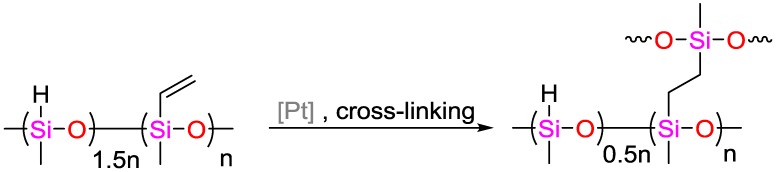
Solidification by crosslinking of a polysiloxane at 120 °C using Karstedt’s catalyst.

Wang et al. developed a method for synthesizing a highly transparent, durable, superhydrophobic, and nanoporous coating: As a precursor, they used a polysiloxane containing Si–H and vinyl–Si groups as well as a methyl-terminated PDMS as reagents (Figure 46) [134]. Due to the Si–CH_n_ groups abundantly present in the polymer, further fluorination to enhance the hydrophobicity is not required, enabling economically friendly production by phase-separation. Due to its physical and mechanical properties, the siloxane-coating may possibly be applied in windshields, safety goggles, etc. 

Polysiloxanes and derivatives are susceptible to fouling due to bacterial adhesion on the hydrophobic surface. Therefore, for bioapplications, it is necessary to apply coatings to the polysiloxanes’ surfaces by chemical modification of grafting. [135,136,137,138] Nguyen and coworkers proposed the grafting of polysilazane PSZ with poly(ethylene oxide) PEO by hydrosilylation using Karstedt’s catalysts [139]. They investigated marine bacteria and found that the grafting density of PEO onto PSZ strongly influenced the bacterial adhesion. The chain length of PEO played a major role: longer chains exhibited higher anti-fouling activities. Zhang et al. used allyl-carboxybetain as grafting reagent on elastomeric PDMS via a Karstedt-catalyzed hydrosilylation to increase the anti-fouling properties. Their protein/bacterial adhesion indicated improved biocompatibility and reduced adsorption and adhesion properties regarding proteins and bacteria [140]. For a systematic description of the influence of long hydrophobic side-chains on antimicrobial activity, the reader is referred to a recent research article on that topic [141].

#### 5.2.2. Printing and Inks

**Figure 47:**
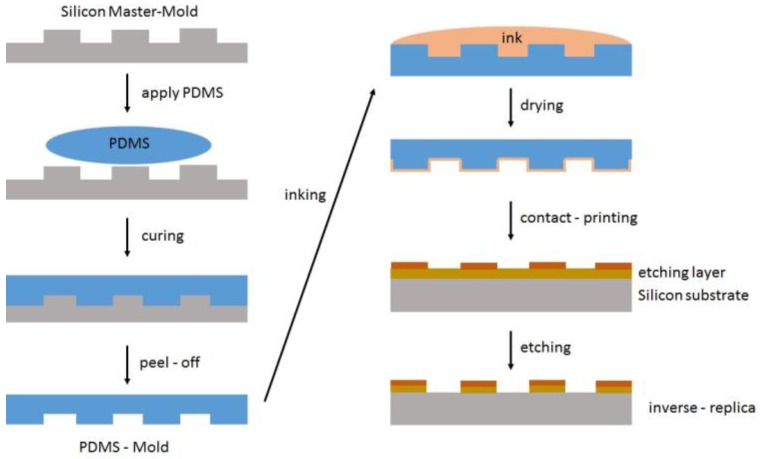
Schematic representation of micro-contact printing for etching processes.

Micro-contact printing is a method to transfer a master mold pattern onto a substrate. The master mold is often created by 2.5-dimensional photolithography (the term “2.5-dimensional” addresses the fact that, by this technique, a surface is generated by the projection of a plane into the third dimension, and that such objects exhibit no overhanging elements despite the fact that they are 3-D). The transfer mold is created by applying a layer of PDMS followed by curing it via hydrosilylation reactions at elevated temperatures to yield an elastomeric PDMS mold (Figure 47) [142]. PDMS (non-crosslinked or only moderately crosslinked) has the disadvantage of swelling in common organic solvents, rendering the necessity of thermal curing. In addition, due to the hydrophobic surface of PDMS, the use of water-based inks containing compounds such as biomolecules or inorganic complexes is challenging [143].

To overcome these disadvantages, Kastner et al. substituted PDMS by functionalized POSS [144]. For functionalization, they used epoxy and carboxylic as well as bifunctional epoxy-carboxylic groups that were covalently attached by platinum-catalyzed hydrosilylations. Epoxy-functionalized POSS could be cured under UV-light under ambient conditions. Masuda et al. developed an amorphous silicon carbide ink for wide-band-gap films to be used in opto-electronics such as LEDs and solar cells [145]. They used cyclopentasilane, cyclohexene and decaborane as source for p-type SiC:H films; however, instead of a classic coating process they applied vapor depositioning by heating the substrate and ink up to 380 °C. After 5 min, the film was approximately 100 nm high, and, depending on the volume ratio of cyclopentasilane to cyclohexene, the band gap *E*_g_ varied from 1.56 to 2.11 eV, while the conductivies ranged from 1.1 × 10^−4^ (semiconductor) to 7.1 × 10^−11^ S/cm (insulator).

Pi et al. prepared solar cells using a silicon-based quantum-dot Si-QD ink [146]. First, Si-QDs were synthesized in a SiH_4_ plasma, then the Si-QDs were hydrosilylated in a solution of octadec-1-ene and mesitylene at 165 °C under argon atmosphere. By printing the Si-QD ink onto the silicon solar cell, the efficiency was increased from 17.2% to 17.5% due to anti-reflective and spectral down-shifting properties. This increase might seem numerically low, but still means a huge economic benefit for the photovoltaic industry. 

#### 5.2.3. Microelectronic Applications

**Figure 48:**
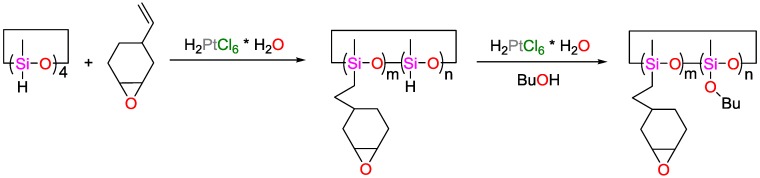
Synthesis of a cycloaliphatic epoxy-silicon hybrid resin by hydrosilylation reactions.

With respect to packaging materials for electronic applications, Gao et al., as well as Zong et al., synthesized cycloaliphatic epoxy-silicon hybrid resins using 1,3,5,7-tetramethylcyclotetrasiloxane and 1,2-epoxy-4-vinyl-cyclohexane as reagents in different compositions (Figure 48). Methyl hexahydrophthalic anhydride was used as curing agent with dodecyl trimethylammonium bromide as accelerant. The fabricated resins showed transmittances >90% at 800 nm and >85% at 400 nm, as well as 5% decomposition temperatures >330 °C and water absorption of less than 2% within the first 12 h in boiling water. UV- and temperature-mediated (120 °C) ageing tests showed no yellow coloring of the samples, rendering these materials candidates for LED-packaging and electronic sealings [147,148,149].

**Figure 49:**
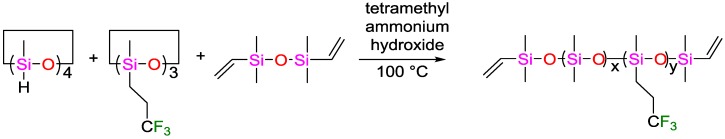
Synthesis of vinyl end-functionalized polysiloxanes.

Contrary to sealing with low electrical conductivity, polymer-derived ceramics offer great potential for application in micro-electrical systems as they exhibit higher conductivities [150,151]. Dalcane et al. performed the hydrosilylation of allylhydrido-poly(carbosilane) SMP10^®^ with divinylbenzene followed by pyrolysis at 800–1400 °C, yielding ceramics with electrical conductivities ranging from 10^−6^ S·cm^−1^ (semiconductor) to 1 S·cm^−1^ (conductor) [152]. Electroactive polymers can undergo actuation if they are exposed to an electric field. Dascalu et al. developed a vinyl end-functionalized polysiloxane from 1,3,5-tris(3,3,3-trifluoropropyl)-1,3,5-trimethylcyclotrisiloxane and octamethylcyclotetrasiloxane (Figure 49) [153], yielding an elastomer with strains of break of up to 850%, a maximum dielectric permittivity of ε′ = 6.4, and an actuation strain of 5.4% at 7.8 V·(µm)^−1^. Although the commercially available acrylic foil VHB-4905 (ε′ = 4.4) exhibits higher maximum achievable actuation strain and dielectric breakdown, the newly developed siloxane shows higher actuation strain within the desired 24 V range. With further research on upscaling and mechanical properties, this siloxane is a promising candidate for actuators and sensors. 

For the increase of dielectric permittivity, Madsen et al. [154] proposed the incorporation of high dielectric permittivity molecules such as 1-ethynyl-4-nitrobenze followed by hydrosilylation to receive a dielectric elastomer. Using a PDMS spacer (that inherently offers high elastic properties) and 5.6 wt % of 1-ethynyl-4-nitrobenze (that raises the dielectric permittivity by 70%), the dielectric elastomer can be used in the field of transducers.

## 6. Conclusions

Hydrosilylation is a key strategy for the synthesis of various organosilicon substrates for functionalized products (or precursors thereof). By careful choice of the catalyst, diastereomerically pure products can be synthesized in high yields. Commonly, these catalysts are platinum-based; recent developments have addressed metal catalysts based on (comparably) inexpensive nickel, iron, and cobalt compounds. Metal-free catalysts such as Lewis acids have also been developed. Notably, stimuli other than heat (such as the application of UV light) can be utilized to start the hydrosilylation in a formulated reaction mixture. For the industrial exploitation of this reaction, adequately-priced catalysts have to be tailor-made to suit the industrial process: High-tech polymers and organofunctional silanes mainly require highly selective catalysts which can be easily recovered. An analysis of potential applications of materials derived from hydrosilylation revealed the fields of base chemicals, coatings and electronics. 

Despite the progress of the recent years, challenges exist to be overcome for industrial applications. In our opinion, the five approaches listed hereinafter inherently bear a huge potential to advance the applicability of the hydrosilylation:

- Low-cost catalysts: Non-platinum transition metal catalysts have drastically lower the catalyst prices compared to platinum, and can be used in very low catalyst loadings.
- Non-metal catalysts: In addition to their cost efficiency, catalysts such as borane-based Lewis acids can be easily separated from the final (polymeric) products. They meet demands of the area of microelectronics, in which traces of metals may cause issues of performance.
- Triggered hydrosilylation: Stimuli other than heat give further flexibility to the processing schedule and eventually enhance the storage stability. Light is a favorable stimulus, as it allows for spatiotemporal control and specific activation.
- Solvent-free processes: These hydrosilylation reactions represent a big step towards green chemistry.
- Selectivity: Chemoselective hydrosilylation can pave the way to novel materials from a broad range of different substrates.

Hence, at the beginning of the second half of its first century, hydrosilylation reactions have advanced to a level that renders them predestined for transfer into commercial products. With ongoing scientific developments, this transfer is likely to occur in the very near future.
